# The complete mitochondrial genome of *Solenocera crassicornis*

**DOI:** 10.1080/23802359.2016.1192498

**Published:** 2016-07-10

**Authors:** Yiming Yuan, Yutao Qin, Shouhai Liu, Xiao Ji, Lihua Xia

**Affiliations:** a East China Sea Environmental Monitoring Center of State Oceanic Administration, Shanghai, China;; b Key Laboratory of Integrated Monitoring and Applied Technology for Marine Harmful Algal Blooms, SOA, Shanghai, China

**Keywords:** Mitochondrial genome, penaeidea, *Solenocera crassicornis*, Solerocerinae

## Abstract

In this study, the complete mitochondrial genome (mitogenome) of the *Solenocera crassicornis* was amplified and analyzed. The mitogenome is 15,946 bp in length, encoding the standard set of 13 protein-coding genes, 22 tRNA genes and two rRNA genes. The overall A + T content is 67.67%; nucleotide frequency of the mitogenome is as follows: A, 32.00%; C, 11.14%; G, 21.19%; and T, 35.67%. There were 16 non-coding regions throughout the mitogenome of *S. crassicornis* ranged from 1 to 1,005 bp in size, of which the largest was between *tRNA^Ile^* and *12S rRNA.* The complete mitogenome sequence information of *S. crassicornis* would play an important part for further studies on molecular systematics and phylogeny.

*Solenocera crassicornis*, an important economic shrimp species, naturally distributes in East China Sea and South China Sea. Meanwhile, it is scattered over India, Singapore and Indonesia. It is well known that mitochondrial DNA information could provide the basic information for the studies in population genetics. In this study, the complete mitochondrial genome (mitogenome) of *S. crassicornis* was first determined. Our result provided essential data in the studies of population genetics and phylogenetic analysis of this species.

In this study, the samples of *S. crassicornis* were collected from the East China Sea (123°03′47″E, 31°42′18″N). The specimen was stored in Sample Room of East China Sea Environmental Monitoring Center of State Oceanic Administration, and its accession number was B0050-1. Fresh tissues were extracted from the muscle of adult individuals. Partial sequences of *COI* and *16S rRNA* genes were amplified by PCR using universal primers. Based on these two PCR products, we designed two specific primers pairs to amplify two DNA fragments ∼5 and 10 kb of the mitogenome of *S. crassicornis*, respectively. Subsequently, these two PCR products were sequenced using Shot Gun Sequencing (Batzoglou et al. 1999). Then all the sequences obtained were analyzed and assembled using the software BioEdit (Hall 1999). The location of tRNAs were determined by using tRNA scan-SE 1.21 (Schattner et al. 2005). Finally, the complete mitogenome sequence of *S. crassicornis* was deposited in the GenBank database under accession number of KU899137.

The length of *S. crassicornis* complete mitogenome was 15,946 bp, consisting of 13 protein-coding genes, 22 tRNA genes, two rRNA genes and 16 non-coding regions. The base composition was A + T bias with the value of 67.67%. Nucleotide frequency of the mitogenome is as follows: A, 32.00%; C, 11.14%; G, 21.19%; and T, 35.67%. The *tRNA^Gln^*, *tRNA^Cys^*, *tRNA^Tyr^*, *ND5*, *tRNA^Phe^*, *tRNA^His^*, *ND4*, *ND4L*, *tRNA^Pro^*, *ND1*, *tRNA^Leu^*, *16S rRNA*, *tRNA^Val^*, and *12S rRNA* were encoded on the light strand of the mitogenome, whereas the remaining genes were encoded on the heavy strand. Totally, eight protein-coding genes (*COII*, *ATP6*, *COIII*, *ND3*, *ND4*, *ND4L*, *CYTB* and *ND1*) started with the ATG typical initiation codon. *ND2* and *ND6* genes shared the ATT initiation codon, while *COI*, *ATP*8 and *ND*5 have the ACG, ATC and GTG initiation codons, respectively. Thirteen complete stop codons were used in the protein-coding genes, of which *ND*2, *COIII*, *CYTB* and *ND1* have stop codon of TCT, TAG, TTA and TTT, respectively, while the remaining genes have the TAA stop codon. There were 16 non-coding regions throughout the mitogenome ranged from 1 to 1,005 bp in size, with the largest one between *tRNA^Ile^* and *12S rRNA*.

To analysis the phylogenetic relationship among typical shrimps (including *S. crassicornis*, nine other shrimps of Penacidae, and one shrimp of Sergestidae), a phylogenetic tree was constructed using Neighbor Joining method by the software MEGA5.1 (Tamura et al. 2011). The phylogenetic tree deriving from amino acid sequences of all protein-coding genes in shrimps was shown in Figure 1. Shrimps of Penaeus clustered first (including *Penaeus*, *Fenneropenaeus*, *Marsupenaeus*, *Litopenaeus*, *Metapenaeopsis*, *Metapenaeus* and *Parapenaeopsis*), and then clustered to *S. crassicornis* of Solerocerinae. *Acetes chinensis* is a single branch in the phylogenetic tree, which belong to Sergestidae. The phylogenetic result constructed by the mitochondrial amino acid sequence is consistent with the evolutionary status of these species. This study would provide basic data for genetic analyses in the future study.

**Figure 1.**
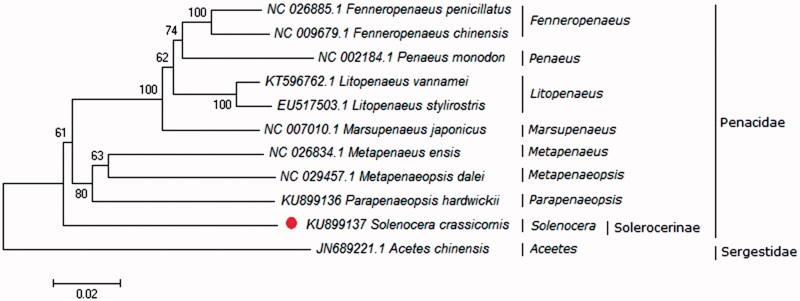
Neighbor Joining tree of the amino acid sequences of all protein-coding genes in shrimps. The numbers on the nodes show the bootstrap percentages.
